# Methylene Blue Administration Reduces Acute Kidney Injury After Living Donor Liver Transplantation: A Single‐Center Retrospective Study

**DOI:** 10.1002/hsr2.72025

**Published:** 2026-03-08

**Authors:** Akira Katayama, Ezeldeen Abuelkasem, Yaroslava Longhitano, Raymond M. Planinsic, Austin C. Smith, David W. Wang

**Affiliations:** ^1^ Department of Anesthesiology and Perioperative Medicine University of Pittsburgh Pittsburgh PA USA; ^2^ Department of Anesthesiology and Resuscitology Okayama University Graduate School of Medicine, Dentistry and Pharmaceutical Sciences Okayama Japan

**Keywords:** acute kidney injury, liver transplantation, methylene blue, reperfusion injury

## Abstract

**Background and Aims:**

The efficacy of methylene blue (MB) in preventing acute kidney injury (AKI) in liver transplant recipients remains unclear. In this study, we hypothesized that pre‐reperfusion administration of MB decreases the incidence of postoperative AKI in living donor liver transplantation (LDLT).

**Methods:**

We retrospectively analyzed data from 415 patients who underwent LDLT between January 2018 to June 2023. The MB group received a bolus of 1–1.5 mg/kg of MB just prior to graft reperfusion, and the control group did not receive MB. The primary outcome was the incidence of postoperative AKI for each stage, as defined by the Kidney Disease Improving Global Outcomes guidelines. Secondary outcomes included post‐reperfusion hemodynamics, hospital length of stay (LOS), the incidence of early allograft dysfunction (EAD), and mortality at 30‐days, 6‐months, and 1‐year.

**Results:**

The incidence of postoperative AKI all stage and AKI stage 1 was significantly lower in the MB group compared to the control group (AKI all stage: 40.8% vs. 30.0%, *p* = 0.049, AKI stage 1: 34.7% vs. 23.3%, *p* = 0.03). In multivariable analysis, MB administration was independently associated with the incidence of AKI stage 1 (OR 0.59, 95% CI 0.36‐0.99, *p* = 0.046). Hemodynamics during post‐reperfusion period were similar among the groups. The incidence of EAD is significantly lower in MB group compared to control group, but hospital LOS and mortality at 30‐days, 6‐months, and 1‐year were similar in both groups.

**Conclusion:**

The administration of MB just prior to graft reperfusion was associated with decreased incidence of postoperative AKI stage 1 in LDLT. MB administration was also associated with reduced incidence of EAD.

## 1 Introduction

Acute kidney injury (AKI) is one of the most common complications after liver transplantation (LT). The incidence of AKI is varied among reports, ranging from 21.6% to 72% [1, 2, 3]. AKI has a negative impact on outcomes, including prolonged intensive care unit and hospital length of stay (LOS), decreased graft survival, and mortality [4]. Previous studies have identified several risk factors for post‐LT AKI, such as obesity, high model for end‐stage liver disease (MELD) score, diabetes mellitus (DM), large volume blood loss, and intraoperative hypotension [5, 6, 7, 8, 9]. In LT, intraoperative hemodynamic instability is often seen, especially after allograft reperfusion, also known as postreperfusion syndrome (PRS), which has been associated with post‐LT AKI [10].

Methylene blue (MB), an inhibitor of vasodilatory effects of nitric oxide (NO) on the endothelium and vascular smooth muscle, has been found to improve hypotension in various clinical situations [11, 12]. Furthermore, MB's positive effect on hemodynamics has been shown to reduce the incidence of AKI in patients who underwent cardiac surgery and critically ill patients [13, 14, 15]. In the setting of LT, MB has been used to prevent and treat PRS and vasoplegia [16].

However, the efficacy of MB in preventing AKI in LT remains unclear. We performed a retrospective study to investigate the efficacy of MB on the incidence of AKI after living donor LT (LDLT). We hypothesized that pre‐reperfusion administration of MB is associated with decreased incidence of postoperative AKI by mitigating hypotension during reperfusion in LDLT.

## 2 Materials and Methods

We retrospectively reviewed the medical records of patients who underwent LDLT between January 2018 and June 2023 at University of Pittsburgh Medical Center (Pittsburgh, USA). All patients who underwent LDLT surgery were enrolled in this study. Patients aged < 18 years, those who had chronic kidney disease dependent on hemodialysis, and those with incomplete data were excluded. This study was approved by the University of Pittsburgh institutional review board (STUDY20050148) and conducted in accordance with the Declaration of Helsinki. The need for individual patient consent was waived by the institutional review board for this minimal‐risk retrospective review.

### 2.1 Data Collection

The patient data were obtained from the electronic medical records. The following preoperative factors were evaluated: sex, age, body mass index (BMI), etiology of end‐stage liver disease, MELD score, laboratory values evaluated within 1 week before surgery, and morbidity of hypertension and DM. Intraoperative data, including the amount of administered fluid and transfusion, blood loss, and the duration of surgery, were recorded as operative factors. Donor information including age, graft weight, graft‐to‐recipient weight ratio (GRWR), cold ischemic time (CIT), and warm ischemic time (WIT) were also retrieved from the electronic medical records. In the present study, patients were divided into 2 groups: patients in the MB group received 1–1.5 mg/kg intravenous bolus of MB mixed with 100 mL of normal saline just prior to reperfusion, and patients in the control group were not. In our institution, administration of MB prior to graft reperfusion was generally recommended as part of the intraoperative protocol. However, the actual use of MB was at the discretion of each faculty anesthesiologist. The postoperative data included the incidence of AKI, the incidence of PRS, mortality at 30‐days, 6‐months, and 1‐year, hospital LOS, the incidence of early allograft dysfunction (EAD), and ventilation time. Hemodynamic parameters including mean arterial pressure (MAP), central venous pressure (CVP), cardiac index, and systemic vascular resistance index (SVRI) were also evaluated. Hemodynamic parameters from 5 min prior to reperfusion to 30 min after reperfusion at 5 min intervals were collected from the anesthetic records.

### 2.2 Study Definition

Postoperative AKI was defined using serum creatinine criteria according to the Kidney Disease Improving Global Outcomes (KDIGO) guidelines [17]. The most recent serum creatinine level within 7 days prior to surgery was set as the baseline creatinine. According to the KDIGO criteria, AKI stage 1 was defined as an increase in serum creatinine of 0.3 mg/dL within 48 h or a rise from 1.5 to 1.9 times baseline within 7 days, AKI stage 2 was defined as a rise from 2.0 to 2.9 times baseline within 7 days, and AKI stage 3 was defined as 3.0 times baseline or more within 7 days after LT. According to the definition by Aggarwal, PRS is defined as a decrease of mean arterial pressure greater than 30% below the value before reperfusion during the anhepatic phase that lasts for at least 1 min, occurring within 5 min of reperfusion of the liver graft [18]. EAD was defined as the presence of one or more of the following variables: bilirubin ≥ 10 mg/dL on postoperative day (POD) 7, or international normalized ratio (INR) ≥ 1.6 on POD 7 according to the Olthoff criteria [19].

### 2.3 Outcomes

The primary outcome was the incidence of each stage of AKI and AKI, including all stages according to the KDIGO guidelines [17]. Secondary outcomes were the incidence of PRS and EAD, hospital LOS, ventilation time, and mortality at 30‐days, 6‐months, and 1‐year. Post‐reperfusion hemodynamics of MAP, CVP, cardiac index, and SVRI were also included in the secondary outcomes.

### 2.4 Statistical Analysis

The sample size was calculated based on the incidence of post‐LT AKI reported around 40% [1]. We considered a reduction of the incidence of AKI from 40% to 25% to be clinically significant. In our institution, approximately 75% of LT recipients received MB just prior to reperfusion. Accordingly, the sample size was calculated to have an 80% power at two‐sided 0.05 significance level. Descriptive statistics are present as frequency (percentages), median with interquartile range (IQR) or mean ± SD, as appropriate. Continuous variables were analyzed using Student's t‐test for normal distribution variables or Mann‐Whitney U test for the variables that were not normally distributed. Categorical variables were compared using the Pearson's chi‐square test. To identify the independent risk factors for AKI, logistic regression model was applied for univariable and multivariable analyses, and odds ratios (ORs) and 95% confidence intervals (CI) were calculated. Repeated measures ANOVA were applied to post‐reperfusion hemodynamic parameters. A *p* value < 0.05 was considered statistically significant. All analyses were conducted using StataSE version.17.0 (College Station, TX, USA) and EZR (Saitama Medical Center, Jichi Medical University, Saitama, Japan), a graphical user interface for R version 4.4.1 (R Foundation for Statistical Computing, Vienna, Austria).

## 3 Results

A total of 421 patients underwent LDLT during the study period. Of these, 1 patient under 18 years of age, 3 patients with chronic kidney disease dependent on hemodialysis, and 2 patients with missing peri‐operative data were excluded, leaving 415 patients to be included in the analyses. Among these 415 eligible patients, 317 patients were included in the MB group and 98 patients were included in the control group. Patient characteristics and pre‐operative laboratory findings are summarized in Table 1. Age, sex, BMI, etiology, and comorbidity were similar between the two groups. Pre‐operative MELD score was also similar among the groups (MB group vs. control group; 16 (10–20) vs. 17 (10–22), *p* = 0.15). In pre‐operative laboratory findings, only INR showed a significant difference among the groups (1.5 (1.3–1.7) vs. 1.6 (1.4–1.9), *p* = 0.01). Table 2 shows the intraoperative characteristics. There were significant differences in the amount of administered crystalloid (*p* = 0.04), colloid (*p* = 0.01) and intraoperative blood loss (*p* = 0.03). Transfusion variables including red blood cell, plasma, platelet, cryoprecipitate, and blood cell salvage were similar among the groups.

**Table 1:** Patient characteristics and preoperative laboratory findings.

	Control (*n* = 98)	MB (*n* = 317)	*P* value
Age, years	57 [46, 65]	60 [51, 66]	0.07
Sex (Male), *n* (%)	63 (64.3)	189 (59.6)	0.41
Body mass index, kg/m^2^	28.5 [24.9, 34.6]	28.1 [25.2, 32.7]	0.56
Etiology			0.75
NASH, *n* (%)	26 (26.5)	100 (31.6)	
Alcoholic, *n* (%)	20 (20.4)	42 (13.3)	
PSC, *n* (%)	12 (12.2)	43 (13.6)	
HCC, *n* (%)	12 (12.2)	51 (16.1)	
Comorbidity			
Hypertension, *n* (%)	46 (46.9)	136 (42.9)	0.48
DM type II, *n* (%)	36 (36.7)	120 (37.9)	0.51
MELD score	17 [10,22]	16 [10, 20]	0.15
Donor age, years	39 [32, 47]	38 [30, 46]	0.35
AST, U/L	49.5 [32, 69.5]	44 [31, 65]	0.14
ALT, U/L	31 [20, 49]	29 [20, 47]	0.78
Albumin, g/dL	3.3 [2.8, 3.8]	3.3 [3, 3.8]	0.36
Bilirubin, mg/dL	2.3 [1.3, 5]	2.1 [1.2, 3.5]	0.17
Platelet, ×10^3^/µL	86 [60, 145]	96 [67, 142]	0.27
PT INR	1.6 [1.4, 1.9]	1.5 [1.3, 1.7]	0.01
Fibrinogen, mg/dL	225 [177, 326]	251 [191, 321]	0.41
Creatinine, mg/dL	1 [0.7, 1.2]	0.9 [0.7, 1.2]	0.51
eGFR, mL/min/1.73 m^2^	78 [58, 108]	86 [60, 100]	0.93
Sodium, mEq/L	135.5 [133, 139]	136 [133, 139]	0.27

*Note:* Data are presented as median [IQR] or *n*=absolute number (%), as appropriate.

Abbreviations: ALT, alanine transaminase; AST, aspartate aminotransferase; DM, diabetes mellitus; eGFR, estimated glomerular filtration rate; HCC, hepatic cell cancer; MB, methylene blue; MELD, model for end‐stage liver disease; NASH, non‐alcoholic steatohepatitis; PSC, primary sclerosing cholangitis; PT INR, prothrombin international normalized ratio.

**Table 2:** Intraoperative characteristics.

	Control (*n* = 98)	MB (*n* = 317)	*P* value
CIT, min	110 [96, 132]	113 [98, 129]	0.83
WIT, min	24 [22, 27]	24 [22, 27]	0.58
Graft weight, g	857 [750, 963]	900 [780, 1000]	0.22
GRWR	0.99 [0.86, 1.21]	1.06 [0.89, 1.25]	0.18
Duration of surgery, min	546.5 [485, 611]	535 [482, 588]	0.20
Crystalloid, mL	6127.5 [5023, 8154]	6800 [5616, 8430]	0.04
Colloid, mL	1250 [1000, 1750]	1000 [400, 1750]	0.01
RBC, mL	0 [0, 600]	0 [0, 600]	0.62
Plasma, mL	0 [0, 0] 12 patients (12.2%) received plasma	0 [0, 0] 31 patients (9.8%) received plasma	0.45
Platelets, mL	0 [0, 0] 8 patients (8.2%) received platelets	0 [0, 0] 22 patients (6.9%) received platelets	0.70
Cryoprecipitate, mL	0 [0, 0] 9 patients (9.2%) received cryoprecipitate	0 [0, 0] 22 patients (6.9%) received cryoprecipitate	0.49
Blood cell salvage, mL	225.5 [0, 475]	225 [0, 450]	0.53
Blood loss, mL	725 [500, 1000]	600 [450, 900]	0.03
Urinary output, mL	1337.5 [730, 1975]	1230 [750, 1840]	0.72

*Note:* Data are presented as median [IQR].

Abbreviations: CIT, cold ischemic time; GRWR, graft‐to‐recipient weight ratio; MB, methylene blue; RBC, red blood cell; WIT, warm ischemic time.

### 3.1 Primary Outcome

The incidence of AKI in MB group and control group is shown in Table 3. The incidence of AKI all stage and AKI stage 1 was significantly lower in the MB group compared to the control group (AKI all stage; 95 (30.0%) vs. 40 (40.8%), *p* = 0.049, AKI stage 1; 74 (23.3%) versus 34 (34.7%), *p* = 0.03). The incidence of AKI stage 2 and stage 3 were similar between the two groups. Univariable and multivariable analyses to identify the factors independently associated with AKI all stage and stage 1 were performed. In univariable analysis for AKI all stage, methylene blue, preoperative MELD score, preoperative serum albumin level, total volume administered, and the amount of blood loss were considered independent factors associated with the incidence of AKI all stage. In multivariable analysis with these 5 variables adding age and sex, preoperative serum albumin level was independently associated with AKI all stage (OR: 0.62, 95% CI 0.44–0.89, *p* = 0.009), but methylene blue was not (OR: 0.66, 95% CI 0.40–1.08, *p* = 0.10). In univariable analysis for AKI stage 1, methylene blue, preoperative MELD score, sex, preoperative serum albumin level, total volume administered, and the amount of blood loss were considered independent factors associated with AKI stage 1. In multivariable analysis including these 6 variables adding age, the administration of methylene blue (OR: 0.59, 95% CI 0.36–0.99, *p* = 0.046) and preoperative serum albumin level (OR: 0.60, 95% CI 0.41–0.88, *p* = 0.01) were independently associated with the incidence of AKI stage 1 (Table 4).

**Table 3:** The primary and secondary outcomes.

	Control (*n* = 98)	MB (*n* = 317)	*P* value
AKI stage 1, *n* (%)	34 (34.7)	74 (23.3)	0.03
AKI stage 2, *n* (%)	4 (4.1)	19 (6.0)	0.62
AKI stage 3, *n* (%)	2 (2.0)	2 (0.6)	0.24
AKI all stage, *n* (%)	40 (40.8)	95 (30.0)	0.049
PRS, *n* (%)	26 (26.5)	73 (23.0)	0.48
EAD, *n* (%)	27 (27.6)	53 (16.9)	0.02
Ventilation time, hour	2.5 [0, 11]	0 [0, 8]	0.10
Hospital LOS, day	8 [7, 14]	8 [6, 14]	0.09
30‐days mortality, *n* (%)	1 (1.0)	4 (1.3)	0.85
6‐months mortality, *n* (%)	3 (3.1)	17 (5.4)	0.35
1‐year mortality, *n* (%)	6 (6.1)	24 (7.6)	0.63

*Note:* Data are presented as median [IQR] or *n* = absolute number (%), as appropriate.

Abbreviations: AKI, acute kidney injury; EAD, early allograft dysfunction; LOS, length of stay; MB, methylene blue; PRS, post‐reperfusion syndrome.

**Table 4:** Multivariable analysis for the incidence of AKI all stage and AKI stage 1.

Variables	AKI all stage				AKI stage 1			
	Odds ratio	95% CI	P value		Odds ratio	95% CI	*P* value	
Age, years	1.00	0.98–1.02	0.97		1.01	0.99–1.03	0.35	
Sex (male)	1.15	0.73–1.79	0.55		1.50	0.92–2.44	0.10	
MELD score	1.02	0.99–1.06	0.15		1.01	0.98–1.05	0.50	
Albumin, g/dL	0.62	0.44–0.89	0.009		0.60	0.41–0.88	0.01	
Total volume administered, L	1.07	0.98–1.16	0.12		1.02	0.94–1.10	0.59	
Intraoperative blood loss, L	1.38	0.97–1.97	0.07		1.31	0.86–1.99	0.20	
Methylene blue	0.66	0.40–1.08	0.10		0.59	0.36–0.99	0.046	

Abbreviations: AKI, acute kidney injury; CI, confidence interval; MELD, model for end‐stage liver disease.

### 3.2 Secondary Outcomes

**Figure 1:**
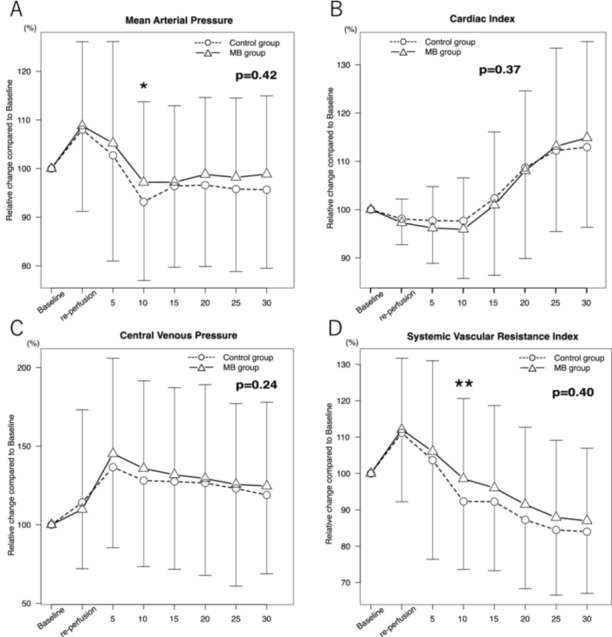
Hemodynamic parameters after reperfusion up to 30 min. Baseline is measured 5 min before reperfusion. Every 5‐min variable measured from reperfusion time up to 30 min after reperfusion was compared with baseline between control group and MB group. (A). MAP, (B). CI, (C). CVP, (D). SVRI. MB, methylene blue; MAP, mean arterial pressure; CI, cardiac index; CVP, central venous pressure; SVRI, systemic vascular resistance index. *: *p* = 0.03, **: *p* = 0.02.

Table 3 also shows the secondary outcomes. Although the incidence of PRS was similar between the two groups (73 (23.0%) vs. 26 (26.5%), *p* = 0.48), the incidence of EAD was significantly lower in the MB group compared to the control group (53 (16.9%) vs. 27 (27.6%), *p* = 0.02). Other secondary outcomes such as hospital LOS, ventilation time, and mortality at 30‐days, 6‐months, and 1‐year showed no significant differences among the groups. Post‐reperfusion hemodynamic parameters are shown in Figure 1. Although MAP and SVRI at 10 min after reperfusion were statistically higher in the MB group than in the control group (*p* = 0.03 and *p* = 0.02, respectively), repeated‐measures ANOVA did not show a significant group effect for MAP, SVRI, cardiac index, or CVP over the entire 30 min post‐reperfusion period. Therefore, MB administration was not associated with a sustained improvement in post‐reperfusion hemodynamics.

## 4 Discussion

In this retrospective study, we analyzed the association between the administration of MB and postoperative AKI in 415 patients who underwent LDLT. To the best of our knowledge, this study is the first to demonstrate the correlation between administration of MB and postoperative AKI in LDLT. We found that administration of MB just prior to re‐perfusion was independently associated with reduced incidence of postoperative AKI stage 1.

Post‐LT AKI is a common complication, and the impact of AKI on post‐LT morbidity and mortality is well recognized. AKI has been associated with prolonged hospital stay, development of chronic kidney disease, reduced graft survival, and increased mortality [1, 4, 19]. Because most factors associated with post‐LT AKI, including obesity, diabetes mellitus, MELD score, donor age, and graft steatosis, are not modifiable at the time of LT [7, 20], identifying intraoperative factors that can reduce development of AKI can have significantly positive impact on postoperative outcomes after liver transplantation.

Since several reports and studies about the efficacy of MB were published [11, 13, 14, 21, 22], MB became widely recognized as a treatment option for vasoplegic syndrome in septic shock, cardiac surgery, and LT. In critically ill patients or those who underwent cardiac surgery, it has been demonstrated that administration of MB reduces the incidence of renal failure [15, 23, 24]. These literatures suggest that administration of MB led to hemodynamic improvement, which resulted in reducing the incidence of renal failure. The reason why MB improves hemodynamics is mainly attributed to the NO and cyclic guanosine monophosphate (cGMP) pathways. NO stimulates guanylate cyclase in endothelial cells and vascular smooth muscle cells to produce intracellular cGMP and cause vascular relaxation. NO is also one of the inflammatory mediators released with reperfusion and is known to contribute to the hypotension seen in PRS [25], and furthermore, it is known that patients with end‐stage liver disease have higher concentrations of NO levels [26]. MB acts as an inhibitor of soluble guanylate cyclase by competing with NO and binding to the heme moiety of the enzyme, preventing cGMP accumulation [27, 28], and MB also directly inhibits NO synthase [29, 30]. Through these mechanisms of NO and cGMP pathways, MB prevents cGMP induced vascular relaxation and restores vascular tone. This is why MB is believed to be effective in mitigating hemodynamics in LT. In our study, MAP and SVRI were higher in the MB group at a single time point (10 min after reperfusion); however, there was no significant overall group effect in the repeated‐measures analysis of post‐reperfusion hemodynamics. This pattern suggests that MB did not produce a consistent or sustained hemodynamic benefit during the 30 min post‐reperfusion period, and the isolated difference at 10 min is unlikely to be clinically meaningful. Accordingly, MB administration was not associated with a sustained improvement in post‐reperfusion hemodynamics in this cohort. Possible reasons for not observing improved post‐reperfusion hemodynamics by MB administration are considered as follows. Firstly, NO levels in patients of this study are unknown. There were many patients in our study that did not have PRS, and it is conceivable that NO concentrations in this population may not be as high. This can affect the results of this study because patients without high concentrations of NO may not benefit as much from MB administration. This was evidenced by no difference in the incidence of PRS between the MB and control groups (23.0% vs. 26.5%). Similarly, previous studies have reported no significant reduction in PRS with MB administration [31], indicating that the evaluation of MB's effect on PRS may require a more uniform patient population, particularly with respect to NO levels. Secondly, several case reports suggest that the effect of MB appears 30 min to 2 h after administration of MB [32, 33]. Because we collected post‐reperfusion hemodynamics only up to 30 min after reperfusion, the comparison of hemodynamics 30 min after reperfusion could not be performed. Thirdly, in animal studies, the effect of MB to ameliorate hypotension was stronger when used as treatment than prophylaxis. Different timing of MB administration may result in different outcomes in hemodynamics.

MB is also known to be able to attenuate renal dysfunction in other ways. In the kidney, NO and cGMP production are associated with lipopolysaccharide‐induced renal proximal tubular cell toxicity [34]. MB's inhibitory effects on NO synthase and cGMP production can result in attenuated renal dysfunction [35]. In addition, MB can also act as an alternative electron acceptor for xanthine oxidase, which can suppress the production of superoxide radicals. MB can be used to prevent free radical damage to the kidney and exhibit renal protective effects [36]. Furthermore, in an animal model, administration of MB reduced pro‐inflammatory factors such as interleukin (IL)‐1β and IL‐18 and increased anti‐inflammatory factors such as IL‐10 and transforming growth factor‐β1, resulting in reducing renal ischemia‐reperfusion injury [37]. Taken together, these renal protective mechanisms of MB, such as inhibition of NO/cGMP signaling, attenuation of oxidative stress, and modulation of inflammatory responses, may partly explain the reduced incidence of AKI stage 1 observed in this study, even in the absence of sustained post‐reperfusion hemodynamic differences.

We also found that serum albumin level was independently associated with the incidence of AKI. The mechanisms of lower albumin level leading to the development of AKI are multifactorial, but one possible explanation is that serum albumin has a renal protective effect by improving renal perfusion, inhibiting apoptosis of renal tubular cells, and promoting the proliferation of renal tubular cells [5, 38, 39]. In fact, multiple studies have identified preoperative lower albumin level as an independent risk factor for AKI in LT [40, 41, 42], and this was consistent with our findings.

In our study, EAD was significantly lower in the MB group compared to the control group. Hepatic ischemia‐reperfusion injury (IRI) influences the prognosis of liver function. The mechanisms of IRI remain unclear, but several factors are known to correlate with IRI, including anaerobic metabolism, mitochondria, cytokines and chemokines, intracellular calcium overload, and oxidative stress [43]. Because MB mitigates oxidative stress as an antioxidant [44, 45], MB administration is expected to ameliorate IRI and postoperative graft function. This theory supports the findings of the current study.

Despite our important findings, there are a few limitations to the current study. First, this was a retrospective, single‐center study. Since MB administration during LT was not randomly assigned, there was potential for selection bias for the administration of MB. Second, patients’ NO levels were unknown in this study. Because of MB's inhibitory effects of the NO/cGMP pathways, comparing patients with different NO levels may affect the results. Third, exact MB administration dosing and timing were not standardized. This lack of standardization may have affected the results. Fourth, the study population included only those who underwent living donor LT. In general, the incidence of PRS is higher in deceased donor LT compared to living donor due to its longer allograft ischemia time. Therefore, it is possible that the deceased donor liver transplant recipient population may yield different results. Fifth, vasopressor use and intraoperative hypotension may act as potential confounders in the relationship between MB administration and postoperative AKI. Although no significant differences were observed in post‐reperfusion hemodynamic parameters between the groups, this retrospective study could not fully account for cumulative vasopressor exposure or the precise duration and severity of hypotension prior to reperfusion. These unmeasured factors represent an important limitation and raise the possibility of residual confounding. Finally, MB administration was independently associated with only AKI stage 1, but not with AKI all stage. However, even AKI stage 1 can lead to increased mortality, graft failure, and CKD [46, 47]. Moreover, in our cohort, the majority of AKI patients were classified into AKI stage 1 (AKI all stage: *n* = 135, AKI stage 1: *n* = 108, 80%). Therefore, we believe our result of independent association between MB administration and AKI stage 1 is clinically important in the setting of LT.

## 5 Conclusion

In conclusion, the present study demonstrates that the administration of MB just prior to reperfusion is associated with a decreased incidence of postoperative AKI stage 1 in LDLT. Furthermore, the administration of MB was also associated with reduced incidence of EAD. Although MB did not improve post‐reperfusion hemodynamics, further large prospective research concerning the effect of MB on the outcome and hemodynamics is warranted.

## Author Contributions

**Akira Katayama:** writing – original draft, writing – review and editing, conceptualization, methodology, data curation, formal analysis, investigation. **Ezeldeen Abuelkasem:** writing – reviewing and editing, methodology, supervision. **Yaroslava Longhitano:** writing – reviewing and editing, methodology. **Raymond M. Planinsic:** writing – reviewing and editing, methodology. **Austin C. Smith:** writing – reviewing and editing, investigation. **David W. Wang:** writing – reviewing and editing, conceptualization, methodology, supervision.

## Funding

The authors received no specific funding for this work.

## Disclosure

All authors have read and approved the final version of the manuscript. David Wen Rui Wang had full access to all of the data in this study and takes complete responsibility for the integrity of the data and the accuracy of the data analysis.

## Conflicts of Interest

The authors declare no conflicts of interest.

## 1 Transparency Statement

The lead author David W. Wang affirms that this manuscript is an honest, accurate, and transparent account of the study being reported; that no important aspects of the study have been omitted; and that any discrepancies from the study as planned (and, if relevant, registered) have been explained.

## Data Availability

The dataset used and/or analyzed for the current study is available from the corresponding author upon reasonable request.
